# Efficacy of Plyometric and TheraBand FlexBar Exercises in Tennis Elbow Patients: A Comparative Study

**DOI:** 10.7759/cureus.61525

**Published:** 2024-06-02

**Authors:** Mohan Kumar G, Nishanthi S, Kamatchi K, Ramachandran S, Sudhakar S, Rajalaxmi V, Veena Kirthika S, Kaushik Raman, Srisaisantoshini Sankaranarayanan, Priya C

**Affiliations:** 1 Musculoskeletal Physiotherapy, Faculty of Physiotherapy, Dr. M.G.R. Educational and Research Institute (Deemed to be University), Chennai, IND; 2 Physiotherapy, Faculty of Physiotherapy, Dr. M.G.R. Educational and Research Institute (Deemed to be University), Chennai, IND; 3 Neurological Physiotherapy, Faculty of Physiotherapy, Dr. M.G.R. Educational and Research Institute (Deemed to be University), Chennai, IND; 4 Cardiovascular and Pulmonary Physiotherapy, Faculty of Physiotherapy, Dr. M.G.R. Educational and Research Institute (Deemed to be University), Chennai, IND; 5 Sports Physiotherapy, Faculty of Physiotherapy, Dr. M.G.R. Educational and Research Institute (Deemed to be University), Chennai, IND; 6 Biotechnology, Dr. M.G.R. Educational and Research Institute (Deemed to be University), Chennai, IND

**Keywords:** exercise therapy, theraband, plyometric, ultrasound, lateral epicondylitis, tennis elbow

## Abstract

Background

Lateral epicondylitis is a common condition involving the arm. It is caused by degenerative changes or overuse of the tendon connecting the elbow joint to the forearm muscle. Plyometric and TheraBand FlexBar (Theraband, Akron, OH, USA) exercises can relieve elbow discomfort, soreness, and weakness. This study examines the effects of plyometric and TheraBand FlexBar exercises with ultrasound on tennis elbow patients.

Methodology

It is an experimental study comprising a total of 30 participants, including individuals of both genders with age groups of 20-40 years were selected by specific criteria for inclusion and exclusion. The participants were randomly assigned into two groups. Group A received plyometric exercises with ultrasound, whereas Group B received TheraBand FlexBar exercises with ultrasound. The outcome measures utilized in this study include patient-rated tennis elbow evaluation (PRTEE) and visual analog scale (VAS) for evaluating the functional disability of the hand, arm, and shoulder.

Results

The results showed a substantial reduction in mean values in Group A compared to Group B, with a p-value of less than 0.001, indicating that plyometric exercises with ultrasound were more effective than TheraBand FlexBar exercises.

Conclusion

Plyometric exercises combined with ultrasound therapy demonstrated significant reductions in discomfort and improvements in function, with plyometric exercises showing superior efficacy compared to TheraBand FlexBar exercises.

## Introduction

The elbow joint's stability and functionality rely on the interplay of bone and soft tissue constraints. While static stabilization is ensured by the osseous framework, dynamic stabilization is facilitated by accompanying muscles, enabling intricate hand movements. Lateral epicondylitis widely noted as tennis elbow is distinguished by painful inflammation at the lateral epicondyle region of the humerus [1]. It affects 1% to 3% of the general population, often impeding daily activities and sports participation due to pain and functional impairment [2].

Lateral epicondylitis arises from overuse or repetitive motions, leading to discomfort and tenderness on the outer portion of the elbow. The primary source of pain and sensitivity in lateral epicondylitis originates from the extensor carpi radialis brevis (ECRB) tendon at the forearm's origin. Full extension of the arms can lead to intense discomfort. Anatomical studies indicate that the ECRB tendon experiences maximum tension during contraction, particularly when the forearms are pronated, the wrist is flexed, and the ulnar deviate (Thesis book: Sadeghzadeh M, "EMG Changes of the Forearm Extensor Muscles at Different Forearm Postures," August 28, 2015; http://hdl.handle.net/10315/30001). This tension is exacerbated by repetitive movements, such as gripping activities and eccentric contractions, which frequently involve the wrist. Consequently, individuals may endure significant pain during these motions [3].

The primary goals of lateral epicondylitis therapy revolve around managing pain, preserving mobility, and strengthening muscles. While various treatment modalities exist, including rest, physical therapy, and pharmacological interventions, the success of these approaches in alleviating pain and improving functional outcomes remains a subject of ongoing investigation [4]. To address these objectives, treatments such as ultrasound therapy and plyometric exercises have been employed effectively.

Ultrasound therapy stands out as one of the utmost commonly employed treatments for alleviating elbow pain. Its benefits extend to pain reduction, inflammation relief, and acceleration of tendon healing processes [5]. Additionally, ultrasound treatment has been observed to contribute to the improvement of grip strength in individuals experiencing elbow discomfort.

In the realm of exercise rehabilitation for tendon disorders, plyometric exercises emerge as a highly effective modality. Plyometrics, also referred to as "plyo" or "jump training," is a form of physical activity that emphasizes forceful and rapid movements involving rapid and powerful Techniques to enhance strength, velocity, and general athletic prowess. The main objective of plyometric exercises is to increase the efficiency of the neuromuscular system by enhancing the stretch-shortening cycle (SSC) in the muscles. The basic principle behind plyometrics is based on the understanding that muscles can generate more force if they are rapidly and forcefully stretched before contracting. This phenomenon is known as the stretch-shortening cycle and can be observed in activities like jumping, sprinting, and throwing. This has shown potential in reducing pain, and inflammation, and improving muscle function in tendon disorders. These exercises, particularly when performed at low speeds, have been found to effectively reduce local inflammation, diminish pain intensity, enhance functional abilities, and even aid in the prevention of tendonitis. Plyometric training aims to enhance power generation by integrating strength and speed of movement. This specialized exercise regimen targets the development of muscles capable of contracting rapidly, facilitating what is commonly known as the stretch-shortening cycle [6].

In addition to plyometrics and ultrasound therapy, there are several other multimodal approaches used In the context of managing tennis elbow, also known as lateral epicondylitis. These approaches often involve a combination of various treatment modalities to address pain, inflammation, and muscle imbalances, and promote tissue healing. The TheraBand FlexBar (Theraband, Akron, OH, USA) serves as a valuable tool for strengthening the muscles affected by tennis elbow. Its use is associated with pain relief, primarily through isolated eccentric strengthening activities, including mobilization and oscillation exercises [7].

A direct comparison of these two interventions in terms of their efficacy in alleviating pain and disability in tennis elbow patients is lacking in the current literature. Therefore, this study looks to address this deficiency by conducting a comparative analysis of plyometric exercises and TheraBand FlexBar therapy with ultrasound treatment. By evaluating the effects of these interventions on pain reduction, functional improvement, and patient-reported outcomes, we seek to provide valuable insights into the optimal management approach for tennis elbow. Additionally, the study aims to explore the mechanisms underlying the efficacy of these interventions, particularly focusing on their effects on tendon healing, muscle strength, and proprioception. Through this comparison study, we aim to contribute to the development of data-based criteria for the management of tennis elbow, Ultimately enhancing the overall well-being of persons afflicted with this incapacitating ailment.

## Materials and methods

Selection of subjects

The study was conducted at the Department of Physiotherapy located in the outpatient section of ACS Medical College and Hospital, Chennai. Ethical approval was obtained from the Institutional Ethics Committee (E-29/PHYSIO/IRB/2023-2024). The study employed an experimental design of a single-blind trial with sample randomization conducted using the fishbowl approach. The study sample size was 30 subjects. Since this study's sample was small, each participant was given to choose a number from 1 to 30 using separate slips of paper. These slips were placed in a container, mixed, and then one slip at a time was randomly selected. The first 15 selected participants were then assigned to an experimental group and the last 15 participants were assigned to a control group. The total of 30 subjects included 13 female and 17 male participants with tennis elbow pain between the ages of 20 and 40. Only individuals who satisfied the specified criteria for inclusion and exclusion were included in this study. The criteria for inclusion were subjects aged 20 to 40 with a visual analog scale (VAS) range between 2 and 6, pain and tenderness in the lateral aspect of the elbow for a duration, not less than 10 days with no history of any previous treatment, positive Mill's and Cozen's tests, and subjects who provided informed consent. The exclusion criteria were subjects with a history of rheumatoid arthritis, severe pain above 7 on the VAS, cutaneous infection, any dislocation of the elbow, and humerus and elbow fractures.

Procedure

Once all participants were thoroughly informed about the protocols, they were given a consent form that had been authorized by the ethics committee. Next, the 30 participants were divided randomly into two cohorts using the fishbowl method. Each cohort consisted of 15 individuals. The participants in this single-blind trial were not aware of which groups they belonged to. Group A was trained with plyometric exercise with ultrasound, whereas Group B was trained with TheraBand FlexBar with ultrasound. The treatment duration for both groups was six weeks. The pre- and post-test measures were done. The elbow evaluation was conducted using a VAS and patient ratings score.

Intervention

Plyometric Exercises With Ultrasound Therapy: For Group A

Group A was given therapeutic ultrasound at a dosage level of 0.8 W/cm^2 ^ at continuous mode for a duration of 10 minutes which was followed by slow progressive plyometric exercises for wrist flexors and extensors and for forearm supinators and pronators for eight repetitions per set with five sets and a interval period of one minute between each set [8]. 

TheraBand FlexBar Exercises With Ultrasound Therapy: For Group B

Group B participants underwent a therapeutic regimen combining ultrasound therapy at the dosage of 0.8 W/cm^2^ in continuous mode for 10 minutes. Following ultrasound, TheraBand FlexBar exercises were administered, five times a week for six weeks. There were three sets of 15 repetitions for the FlexBar workout. The exercise required four seconds to complete each time, with an interval of 30 seconds in between each round of recurrences. The participants assumed a seated position with elbows flexed at approximately 90 degrees, holding the TheraBand FlexBar in the midlevel. TheraBand FlexBar level-1 yellow color, extra light (6 lbs) was used [9].

The exercise routine involved a series of movements: (i) Flexion: Participants twisted the FlexBar from above to bottom to perform wrist flexion; (ii) Extension: Holding the FlexBar in the wrist flexed position, participants have to extend the forearm; (iii) Supination: Using the FlexBars one tip, participants bent it in the direction of supination; (iv) Pronation: Participants grabbed one tip of the FlexBar and bent it in the direction of pronation.

Outcome measures 

Visual Analog Scale 

Equivalent to a numeric rating scale (NRS), a VAS is a straight line, traditionally 10 cm long, with language anchors at either end for instance, "no pain" on the far left and "the most intense pain conceivable" on the opposite right end. The patient symbolizes the line at the exact spot where they would rate their level of pain [10]. 

Patient Rated Tennis Elbow Evaluation

The patient-rated wrist evaluation (PRWE) served as the cornerstone for the patient-rated tennis elbow evaluation (PRTEE), which also stipulated information compiled from a predecessor investigation that reviewed the psychometric attributes of evaluation criteria for subjects having lateral epicondylitis. It included solely five pain and 10 functional categories in the questionnaire design. The pain score, which comprises the combined value of five items, and the physical impairment ratings, leading to the sum of ten components decapitated into two, are attainable and equally weighted and result in a total score out of 100. The PRTEE is a highly sensitive, reproducible, and reputable tool for evaluating lateral epicondylitis [11].

## Results

A comprehensive analysis was conducted on the collected data, employing both descriptive and inferential statistics. SPSS Statistics for Windows, Version 24.0 (IBM Corp., Armonk, NY, USA) was used to examine all parameters. The paired and independent t-tests determined the statistical difference between groups.

Analyzing the post-test mean scores among the VAS shows considerable decreases in groups A and B; however, the group with the lower mean value (Group A, Plyometric exercises with ultrasound therapy) is likely more efficient than the group with the higher mean value (Group B, TheraBand FlexBar exercises with ultrasound therapy) (P≤ 0.001). Therefore, the null hypothesis is refuted (Table 1).

**Table 1:** VAS score comparison among Group A and Group B in pre-test and post-test values. SD, Standard deviation; df, Degree of freedom, VAS, Visual analog scale. Group A, Plyometric exercises with ultrasound therapy; Group B, TheraBand FlexBar exercises with ultrasound therapy.

#Test	#Group A		#Group B		t -Test	df	Significance
	Mean	SD	Mean	SD			
Pre-test	4.86	.915	4.93	1.03	-.187	28	.853
Post-test	1.00	.755	2.00	1.19	-2.73	28	.000

Comparing the mean scores among Group A and B on the PRTEE score reveals a suggestive decrease in the post-test, of both groups' means; however, Group A, Plyometric exercises with ultrasound therapy, is likely more efficient than Group B, TheraBand FlexBar exercises with ultrasound therapy (P≤0.001). Therefore, the null hypothesis is refuted (Table 2).

**Table 2:** PRTEE score comparison among Group A and Group B in pre-test and post-test values. SD, Standard deviation; df, Degree of freedom, PRTEE, Patient-rated tennis elbow evaluation. Group A, Plyometric exercises with ultrasound therapy; Group B, TheraBand FlexBar exercises with ultrasound therapy.

#Test	#Group A		#Group B		t -Test	df	Significance
	Mean	SD	Mean	SD			
Pre-test	3.21	.444	3.34	.594	-.671	28	.508
Post-test	.485	.309	.884	.339	-3.38	8	.000

A difference that is statistically significant in mean scores is seen within Groups A and Group B's VAS and PRTEE scores between the pre-test and post-test at p ≤ 0.001 (Tables 3, 4).

**Table 3:** VAS score comparison within Group A and Group B among pre-test and post-test values. SD, Standard deviation, VAS, Visual analog scale. Group A, Plyometric exercises with ultrasound therapy; Group B, TheraBand FlexBar exercises with ultrasound therapy.

#Group	Pre-test		Post-test		t -Test	Significance
	Mean	SD	Mean	SD		
Group A	4.86	.915	.00	.755	16.53	.000
Group B	4.93	1.03	2.00	1.19	8.87	.000

**Table 4:** PRTEE score comparison within Group A and Group B between pre-test and post-test values. SD, Standard deviation; PRTEE, Patient-rated tennis elbow evaluation. Group A, Plyometric exercises with ultrasound therapy; Group B, TheraBand FlexBar exercises with ultrasound therapy.

#Group	Pre-test		Post-test		t-Test	Significance
	Mean	SD	Mean	SD		
Group A	3.21	.444	.485	.309	20.67	.000
Group B	3.34	.594	.884	.339	13.29	.000

## Discussion

A comparison study between plyometric exercise with ultrasound therapy and TheraBand FlexBar exercises with ultrasound for lateral epicondylitis (tennis elbow) revealed a significant level of pain reduction and functional improvement. The findings indicated that plyometric activities combined with ultrasound therapy provided superior outcomes compared to TheraBand FlexBar exercises with ultrasound. Tennis elbow, often stemming from overuse, challenges the tendon's natural healing capacity. Functional abilities were assessed using the PRTEE questionnaire as an outcome measure.

In a Greek study comparing the benefits of low-level laser and plyometric workouts for lateral epicondylitis treatment, plyometric training demonstrated efficacy in reducing symptoms, local inflammation, and pain [8]. The preliminary results disclosed that, both after the completion of the therapy and during the follow-up, the combination of laser and plyometric activities was a more effective treatment than the placebo laser combined with the same plyometric exercises. Further research is required to determine the absolute and relative efficacy of the aforementioned regimen [8].

Similarly, research from Wisconsin highlighted the benefits of plyometric training for the posterior shoulder and elbow, indicating improved power transmission in elbow extensor muscles [12]. Plyometric exercises may thus enhance performance in overhead sports requiring power [12]. A Dutch investigation on electrophysical therapy for medial and lateral epicondylitis effectiveness favored laser therapy over placebo in the short term, while plyometric exercises showed promise in the long term for lateral epicondylitis [13].

In accordance with the recently published study by Novak et al. (2023) plyometric training with resistance band exercises exclusively enhanced lower-body coordination for both horizontal and vertical jumps, but possesses no influence on junior tennis players' first-step quickness, acceleration, speed, change of direction speed, or reactive agility traits [14]. The findings feature valuable insights for coaches to establish an array of tennis-specific scenarios that foster optimal performance, particularly in terms of players' neuromuscular conditioning [14].

Thus with the study results and existing literature evidence plyometric exercises, particularly when combined with ultrasound therapy offer an effective treatment approach for managing elbow pain associated with lateral epicondylitis.

Limitations of the study

The study had a small sample size, which could limit the generalizability of the findings. Additionally, only participants in a specific age bracket were included in this research. The geographical discrepancy was not considered taken into consideration.

Future scope of the study

A larger sample size and a diverse age category would provide more robust results and better, represent the broader population of tennis elbow patients. 

## Conclusions

The study demonstrated substantial pain relief and functional capacity improvement through the implementation of plyometric exercises with ultrasound therapy. Group A which underwent plyometric exercises with ultrasound, exhibited superior effectiveness compared to group B, which engaged in TheraBand FlexBar exercises with ultrasound. These findings concluded that plyometric exercises in conjunction with ultrasound therapy can be the preferred treatment option for managing lateral epicondylitis.
